# Acetylated *Dendrobium huoshanense* polysaccharide: a novel inducer of apoptosis in colon cancer cells via Fas–FasL pathway activation and metabolic reprogramming

**DOI:** 10.3389/fonc.2025.1529868

**Published:** 2025-03-04

**Authors:** Liang Yao, Chen Gu, Ruipeng Ge, Xiaoqian Zhang, Xinqian Meng, Lei Wang, Daiyin Peng, Guozhuan Li

**Affiliations:** ^1^ School of Pharmacy, Anhui University of Chinese Medicine, Hefei, China; ^2^ Anhui Academy of Chinese Medicine, Hefei, China; ^3^ Ministry of Education (MOE)-Anhui Joint Collaborative Innovation Center for Quality Improvement of Anhui Genuine Chinese Medicinal Materials, Hefei, China; ^4^ Anhui Province Key Laboratory for Research and Development of Research and Development of Chinese Medicine, Hefei, China

**Keywords:** *Dendrobium huoshanense* polysaccharide, apoptosis, Fas–FasL signaling pathway, anti-tumor mechanism, metabolic reprogramming

## Abstract

**Introduction:**

Not all polysaccharides function as antitumor drugs, nor do they universally possess the same advantages regarding safety and biocompatibility. Those polysaccharides that are effective antitumor agents typically demonstrate superior safety profiles and biocompatibility compared to synthetic anticancer drugs, which can exhibit high toxicity and harmful side effects. *Dendrobium huoshanense* polysaccharide (DHP) has been recognized for its potential bioactive properties, particularly in anti-tumor treatment. This study investigates the effects of DHP on the proliferation and apoptosis of HCT116 colon cancer cells.

**Methods:**

DHP was extracted according to previously published experimental methods. The inhibitory effects of DHP were evaluated using IEC6, Caco-2, and HCT116 cell lines, with changes in cell morphology observed via transmission electron microscopy. After establishing the conditions for DHP administration, flow cytometry was employed to assess its effects on apoptosis, reactive oxygen species (ROS), and mitochondrial membrane potential of HCT116 cells. Additionally, immunoprecipitation, quantitative real-time polymerase chain reaction (qRT-PCR), Western blotting, and biomarker detection were utilized to investigate the mechanisms underlying DHP’s inhibition of HCT116 cells and its impact on metabolic reprogramming.

**Results:**

In the present study, we observed that DHP treatment at 600 μg/ml for 24 h reduced HCT116 cell viability to 54.87%. In contrast, the inhibitory effect of DHP on the viability of IEC6 and Caco-2 cells was relatively mild. The specific mechanism involves DHP activating the mitochondrial apoptotic pathway leading to the downregulation of key metabolic intermediates and enzymes such as uridine diphosphate N-acetylglucosamine (UDP-GlcNAc) and ST6Gal-I. By inhibiting ST6Gal-I activity, DHP activates the Fas/FasL signaling pathway. Additionally, DHP-induced ROS production effectively triggers apoptosis in HCT116 cells.

**Conclusion:**

Our study demonstrates that DHP effectively inhibits the proliferation and induces apoptosis in HCT116 colon cancer cells through the activation of the Fas–FasL signaling pathway and metabolic reprogramming. The selective inhibitory effect of DHP on HCT116 cells, the activation of both death receptor and mitochondrial apoptotic pathways, and the modulation of metabolic reprogramming provide novel insights into the potential therapeutic strategies for colon cancer.

## 1 Introduction

Colorectal cancer (CRC) is among the most prevalent malignant tumors. A clinical study projects that by 2023, approximately 153,020 individuals will be diagnosed with CRC (1). Colorectal resection and chemotherapy are common treatments for colorectal cancer; however, they are accompanied by side effects and safety concerns (2, 3). Hence, developing new drugs with slight or no side effects for CRC treatment is crucial.

Although most polysaccharides are primarily used for structural support and energy storage, certain polysaccharides with specific structures exhibit significant biological activity (4, 5). Numerous studies have shown that certain polysaccharides with special structures inhibit the proliferation of tumor cells potently by depressing tumor angiogenesis, inducing tumor cell apoptosis, or regulating the immune system, boosting anti-tumor immune responses (6, 7). *Astragalus* polysaccharides, polysaccharides extracted from the plant *Astragalus membranaceus*, have shown therapeutic potential in anti-colorectal tumors by regulating STAT3/galectin-3/LAG3 signaling pathway (8). Moreover, polysaccharides exhibit no or slight side effects on normal cells compared with chemotherapy drugs. Based on the above discussion, the potential role of polysaccharides in the treatment of colorectal neoplasms deserves further exploration.

Apoptosis is a universal form of programmed cell death, a significant phenomenon for cells functioning regularly (9, 10). Tumor cells possess varying degrees of apoptosis resistance; therefore, activating the apoptosis program in tumor cells is crucial for anti-tumor therapy. The Fas receptor–Fas ligand (Fas–FasL) signaling pathway is a vital modulator of tumor cell apoptosis (11, 12). Combined Fas and FasL trigger apoptosis in tumor cells (13). Fas trimer recruits Fas-associated death domain protein, which activates the cascade of apoptosis-associated caspase triggering apoptosis (14). Notably, the Fas–FasL signaling pathway reportedly has a highly complex function in CRC. First, in the preliminary stage of CRC, aberrant activation of the Fas–FasL signaling pathway aggravates ulcerative colitis. Similarly, in the advanced state of CRC, the Fas–FasL signaling pathway significantly influences immunological escape and apoptosis resistance (15–17). Consequently, modulating the Fas–FasL signaling pathway in CRC is crucial.

*Dendrobium huoshanense*, a valuable Chinese medicinal herb, is predominantly found in Huoshan and its surrounding regions in China and is highly prized for its rich medicinal properties. As a major bioactive component, *Dendrobium huoshanense* polysaccharide (DHP) is a water-soluble polysaccharide, which is composed of mannose and glucose in a molar ratio of 75.81:24.19 after a series of separation and purification (18). It has demonstrated to possess significant biological activities, including antioxidant and anti-colitis effects, highlighting its potential as a novel natural medicine (19, 20). DHP reportedly thwarts CRC cell proliferation. However, the anti-tumor mechanism of DHP remains elusive necessitating further investigation (21–23). Considering the metabolism regulatory activity of polysaccharides in various metabolic disturbance syndromes, such as diabetes, and the ubiquitous metabolic disturbance in cancer cells (24, 25), it has been well documented that acetylation modification can enhance the water solubility and anti-tumor activity of polysaccharides (26). In our previous study, we found that DHP is an acetylated polysaccharide (18). This study hypothesized that DHP could induce apoptosis in HCT116 cells via activation of the Fas–FasL signaling pathway and modulation of metabolic reprogramming. To test this hypothesis, we systematically evaluated the effects of DHP on the viability of normal intestinal epithelial IEC6 cells and two colorectal cancer cell lines, Caco-2 and HCT116, to determine whether the inhibitory effect of DHP is selective. Subsequently, we conducted an in-depth investigation into the effects of DHP on apoptosis and metabolic alterations in HCT116 cells. Specifically, we examined the activation of the Fas–FasL pathway by assessing the expression levels of relevant proteins and investigated the role of reactive oxygen species (ROS) in apoptosis induction. Furthermore, we explored the impact of DHP on key metabolic intermediates, such as UDP-GlcNAc, and enzymatic activities, like those of ST6Gal-I, which play crucial roles in metabolic processes.

## 2 Materials and experimental design

### 2.1 Materials

*Dendrobium huoshanense*, originating from Huoshan County, Anhui Province, was authenticated by Professor Nianjun Yu of the Anhui University of Traditional Chinese Medicine. HCT116, Caco-2, and IEC-6 cell lines and their specialized medium were purchased from Wuhan Procell Life Science and Technology Co., Ltd. N-acetyl cysteine (NAC, purity >98%) and GSK-690693 (purity 98%) were purchased from Beyotime Biotechnology. Cell counting kit-8 (CCK-8 kit) was acquired from Biosharp Science and Technology Co., Ltd. The antibodies included Fas, Fas-associated death domain protein (FADD), caspase-3, Bax, Bcl-2, phosphatidylinositol 3-kinase (PI3K), protein kinase B (AKT), and p-AKT and were offered by Wuhan Sanying Biotechnology Co., Ltd. (1:5,000, China). All other chemicals mentioned in this study were of analytical grade.

### 2.2 Experimental design

#### 2.2.1 Isolation and purification of DHP

The isolation and purification methods, along with the physicochemical properties and characterization of DHP, are detailed in accordance with our previously published studies (18, 19). Briefly, at room temperature, a 95% ethanol mixture was stirred for 2 h to extract lipids and pigments, after which the ethanol was removed, and the powder was dried overnight. The dried powder was extracted with ultrapure water at 65°C and then mixed with four volumes of ethanol (95%, v/v). The mixture was stored at 4°C overnight before being centrifuged at 4,000 rpm for 15 min to remove proteins from the precipitate using the Sevag method. Subsequently, impurities with a molecular weight less than 3,500 Da were removed by dialysis to obtain DHP (depolymerized hydrolyzate of polysaccharides). Then, DHP was purified through a DEAE-52 cellulose column (1.6 × 60 cm) and eluted with water and sodium chloride (0–1.0 M). Active fractions were screened under the guidance of *in vitro* antioxidant activity and further eluted with ultrapure water on a Sephacryl S-300 column (1.6 × 70 cm). The major active fractions were collected for subsequent experiments.

#### 2.2.2 Cell culture

HCT116 cells and Caco-2 cells are distinct colorectal cancer cell lines, while IEC6 cells are a normal intestinal epithelial cell line. All cell lines were cultured in a McCoy’s 5A (Procell, China) or Roswell Park Memorial Institute 1640 medium (Biosharp, China) with 1% penicillin–streptomycin (Beyotime, China) and 10% fetal bovine serum (FBS, Beyotime, China). They were put in a cell incubator at 37°C in an atmosphere of 5% CO_2_.

#### 2.2.3 Cell viability assay

Cell counting kit-8 (CCK-8) was applied to evaluate the cell viability. HCT116, Caco-2, and IEC-6 cells were inoculated at 5 × 10^5^ cells/ml in a 96-well plate overnight. The culture conditions for various cell lines are detailed in the “Cell Culture” section. Subsequently, DHPs (0, 50, 100, 200, 400, 600, and 800 μg/ml) were added for 12, 24, and 48 h, followed by the addition of a CCK-8 solution and incubation for 40 min at 37°C. Ensuing measurements were made by gauging the absorbance at 450 nm using a microplate reader (SpectraMax i3X, MD, USA).

#### 2.2.4 Morphological analysis

Ordinary optical microscopes and transmission electron microscopes (TEM, JEM-2100, TFS, USA) were used to analyze the morphological features of HCT116 cells. HCT116 cells were seeded at 4 × 10^5^ cells/ml in a six-well plate overnight. The normal group was not treated with DHP; the normal group did not receive any DHP treatment. The treatment group was administered 600 μg/ml of DHP. The control group received both 600 μg/ml of DHP and 20 μmol/ml of KR-33493, a Fas inhibitor. After 24 h of cultivation, the cells were collected and fixed overnight in 2.5% glutaraldehyde. The samples were post-fixed in 1% osmium tetroxide in a cacodylate buffer for 90 min at 0°C, followed by washing, dehydration, and embedding in Epon-Araldite resin. Serial ultrathin sections (30–40 nm) were collected onto copper grids and finally stained with 0.5% uranyl acetate followed by 1% lead citrate. The ultrastructure analysis of stained sections was observed under a TEM.

#### 2.2.5 Apoptosis analysis

The apoptosis rate was determined using the Annexin V-FITC Apoptosis Detection Kit (Beyotime, China). HCT116 Cells were seeded at 4 × 10^5^ cells/ml in a six-well plate overnight, grouped, and treated as described in the “Morphological analysis” section. We incubated the cells with the antioxidant NAC at a concentration of 20 μM and compared them with the DHP-treated group to further investigate whether reactive oxygen species (ROS) is the primary cause of cell death (27). Subsequently, the cell samples were processed following the instructions of the protocol of the Annexin V-FITC Apoptosis Detection Kit. Data were determined using flow cytometry (BD FACSCelesta, BDMDSH, China) and analyzed using FlowJo 10.8.1.

#### 2.2.6 Estimation of the ATP content

The ATP content assay kit (Beyotime, China) was used to determine the ATP content. HCT116 cells were seeded at 4 × 10^5^ cells/ml in a six-well plate overnight, grouped, and treated as described in the “Morphological analysis” section. ATP samples were extracted using a radioimmunoprecipitation assay (RIPA) lysis buffer. Briefly, the cells were lysed on ice for 40 min and centrifuged at 12,000 × *g* and 4°C for 10 min. The supernatants were collected for subsequent gauging. According to the instructions of the ATP content assay kit, we used a microplate reader (SpectraMax i3X, MD, USA) to measure the ATP concentration in the samples.

#### 2.2.7 Measurement of the mitochondrial membrane potential (MMP)

The MMP was evaluated using the JC-1 immunofluorescent (Beyotime, China) staining and flow cytometry. HCT116 cells were planked in a six-well plate, grouped, and treated as described in the “Morphological analysis” section. Cell samples were collected and processed according to the instructions of the MMP assay kit. Data were determined using flow cytometry (BDFACSCelesta, BDMDSH, China) and analyzed using FlowJo 10.8.1.

#### 2.2.8 Quantification of Intracellular ROS

Intracellular ROS were determined using 2′,7′-dichlorofluorescein diacetate (DCFH-DA, (Beyotime, China). HCT116 cells were cultured in a six-well plate, grouped, and treated as described in the “Morphological analysis” section. We collected cell samples and processed them according to the instructions of the ROS assay kit. DCF was gauged using flow cytometry (BD FACSCelesta, BDMDSH, China) and analyzed using FlowJo 10.8.1.

#### 2.2.9 Determination of the electron transport chain (ETC) complex activity

The mitochondrial complex activity was evaluated using the mitochondrial complex I and IV kits. HCT116 cells were incubated in a six-well plate, grouped as described in the “Morphological analysis” section and treated with DHP for 12 h. Mitochondrial complex samples were extracted using RIPA lysis buffer. Briefly, the cells were lysed on ice for 40 min and centrifuged at 12,000 × *g* and 4°C for 10 min. The supernatants were collected for subsequent experiments. Next, we processed the mitochondrial samples following the instructions of the Mitochondrial Complex I Activity Assay Kit and Mitochondrial Complex V Activity Assay Kit (AmyJet Scientific Inc., China). The absorbances were measured at corresponding wavelengths using a microplate reader (SpectraMax i3X, MD, USA).

#### 2.2.10 Evaluation of caspase-8, Bid/tBid, sialic acid α-2,6-galactose (SA α-2-6 gal), and ST6β-galactosamine alpha-2,6-sialyltransferase 1 (ST6Gal-I) activities

Caspase-8 enzyme-linked immunosorbent assay (ELISA) Kit (AAT Bioquest, USA), Human Cleaved Bid/tBid ELISA Kit (FineTest, China), SA α2-6 gal ELISA Kit (SZKBT, China), and ST6Gal-I ELISA Kit (Thermo Fisher, USA) were used to determine content. The samples were acquired as described in the “Determination of ETC complex activity” section. Subsequently, we processed the samples in accordance with the kits’ instructions and measured the absorbance at the specified wavelength using a microplate reader (SpectraMax i3X, Molecular Devices, USA).

#### 2.2.11 Immunoprecipitation

To further verify the association between α-2-6 sialylation modification on Fas and DHP, proteins were extracted from HCT116 cells that had been incubated with 600 μg/ml of DHP for 24 h. The treatment was terminated by adding ice-cold PBS. The instructions for the immunoprecipitation kit (Beyotime, China) were followed next, and the cells were subsequently centrifuged to remove unbound antibodies and washed twice with ice-cold PBS. Subsequently, cells were lysed in a buffer containing 50 mM Tris-HCl (pH 7.4), 1% Triton X-100, and protease inhibitors on ice for 15 minutes. Lysates were centrifuged, and the supernatant was collected and incubated overnight with 40 µl of prewashed anti-IgM-conjugated agarose beads. Precipitated proteins were eluted from the complexes by boiling in SDS-PAGE sample buffer. Proteins were resolved by SDS-PAGE and immunoblotted for the Fas-binding protein, FADD. The PVDF membrane was stripped using Restore Western Blotting Stripping Buffer, and Fas was re-probed using a chemiluminescence apparatus (Tanon 5200, Tanon, China).

#### 2.2.12 Estimation of lactic acid (LA) and uridine diphosphate N-acetylglucosamine (UDP-GlcNAc) contents

The LA assay kit (NJJCBIO, China) and the Human UDP-GlcNAc ELISA Kit (NEWLF, China) were employed to quantify the respective contents. The idiographic operations were performed according to the instructions in the kit, and the data were measured using a microplate reader (SpectraMax i3X, MD, USA).

#### 2.2.13 Western blotting

Protein samples were prepared as described in the “Determination of ETC complex activity” section. Protein concentration was determined using a bicinchoninic acid (BCA) protein assay kit. Subsequently, equal amounts of protein samples were electrophoresed in 8%–15% gradient sodium dodecyl sulfate-polyacrylamide gel electrophoresis and transferred onto polyvinylidene fluoride membranes. The membranes were blocked with the sealing solution for 40 min and incubated with corresponding primary antibodies overnight at 4°C. Next, the membranes were incubated with a proper secondary antibody and visualized using the Enhanced Chemiluminescent Substrate (Biosharp, China) Western blot detection reagents. Protein expression was quantified through gray-level analysis of bands using the ImageJ software. The housekeeping protein β-actin was considered as the loading control.

#### 2.2.14 Quantitative real-time polymerase chain reaction confirmation

Total RNA was extracted from HCT116 cells using a total RNA extraction kit. cDNA was synthesized using the Prime Script RT Master Mix Kit. qRT-PCR reaction was performed using SYBR Green PCR Master Mix, and the qRT-PCR primers were designed and synthesized by Sangon Biotech, as listed in **Table 1**. Relative mRNA expression levels were evaluated using the 2^−ΔΔCt^ method. Relative mRNA expression levels were detected by qRT-PCR instrument (CFX Touch, Bio Rad, USA).

**Table 1:** Primer sequence.

mRNA	Forward primer	Reverse primer
Fas	CCCAGTCCTTCACTTCTATGTTC	GTAGCACAGTTCAGTCTCGAC
FADD	GTGGCTGACCTGGTACAAGAG	GGTAGATGCGTCTGAGTTCCAT
Caspase-3	ATGGAGAACAACAAAACCTCAGT	TTGCTCCCATGTATGGTCTTTAC
Caspase-8	GAACGAGCATCACTTCCTCATT	GGCATCAACTTCCTTAGAGTCAA
Bax	AGACAGGGGCCTTTTTGCTAC	AATTCGCCGGAGACACTCG
Bcl-2	GAGAGCGTCAACAGGGAGATG	CCAGCCTCCGTTATCCTGGA
ST6Gal-I	AACTCTCAGTTGGTTACCACAGA	GGTGCAGCTTACGATAAGTCTT
PI3K	TTATTGAACCAGTAGGCAACCG	GCTATGAGGCGAGTTGAGATCC
AKT	ATGAACGACGTAGCCATTGTG	TTGTAGCCAATAAAGGTGCCAT
β-actin	CCTAGAAGCATTTGCGGTGG	GAGCTACGAGCTGCCTGACG

#### 2.2.15 Statistics

Statistical analysis was performed using SPSS 23.0 software and Graphics Pad Prism 9.5 software. Data were expressed as mean ± standard deviation (SD), and statistical results were compared using one-way analysis of variance (ANOVA), and two-sample t-test was employed to compare the means between the two groups. A value of p < 0.05 was considered statistically significant, and p < 0.01 was considered significant.

## 3 Results

### 3.1 DHP inhibited cell proliferation in CRC cells

**Figure 1:**
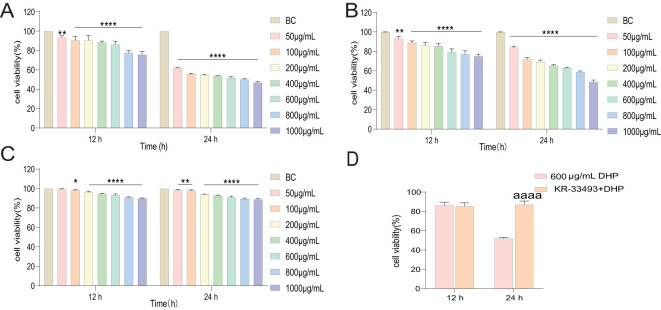
Inhibitory effects of DHP on cells. **(A)** The inhibitory effects on HCT116 cells. **(B)** The inhibitory effects on Caco-2. **(C)** The inhibitory effects on IEC6. **(D)** To evaluate the inhibitory effect of DHP on HCT116 cells following KR-33493 interference, a two-sample t-test was conducted to compare the results. Data are presented as means ± S.E.M., n = 3 (^*^p < 0.05; ^**^p < 0.01; ^****^p < 0.0001 versus BC; ^aaaa^p < 0.0001 versus KR33493 + DHP). BC, base check group; DHP, *Dendrobium huoshanense* polysaccharide.

To preliminarily determine the DHP anti-tumor activity, the cell viability properties of HCT116, Caco-2, and IEC6 cells subjected to DHP and KR-33493 administration were tested using the CCK-8 kit. We observed that 600 μg/ml of DHP significantly inhibited the proliferation of HCT116 cells after 24 h of treatment reducing the cell survival rate to approximately 54.87% (**Figure 1A**). In contrast, DHP weakly inhibited the viability of Caco-2 cells by approximately 37%, while it had a minimal inhibitory effect on the viability of IEC6 cells, reducing it by approximately 8.97%, suggesting that the DHP inhibitory effect might be selective (**Figures 1B, C**). DHP inhibited HCT116 cells in a dose-saturated manner, inconsistent with previous reports (28). To rudimentarily confirm the receptors involved, HCT116 cells were pre-treated with Fas receptor inhibitor KR-33493. The proliferation of the pre-treated (with KR-33493) HCT116 cells was increased compared with that of HCT116 cells treated with DHP only (**Figure 1D**, p < 0.0001). Therefore, we chose 600 μg/ml of DHP for 24-h incubation as the subsequent drug administration condition for cell experiments.

### 3.2 DHP distorted the morphologies of HCT116 cells and their subcellular structures

**Figure 2:**
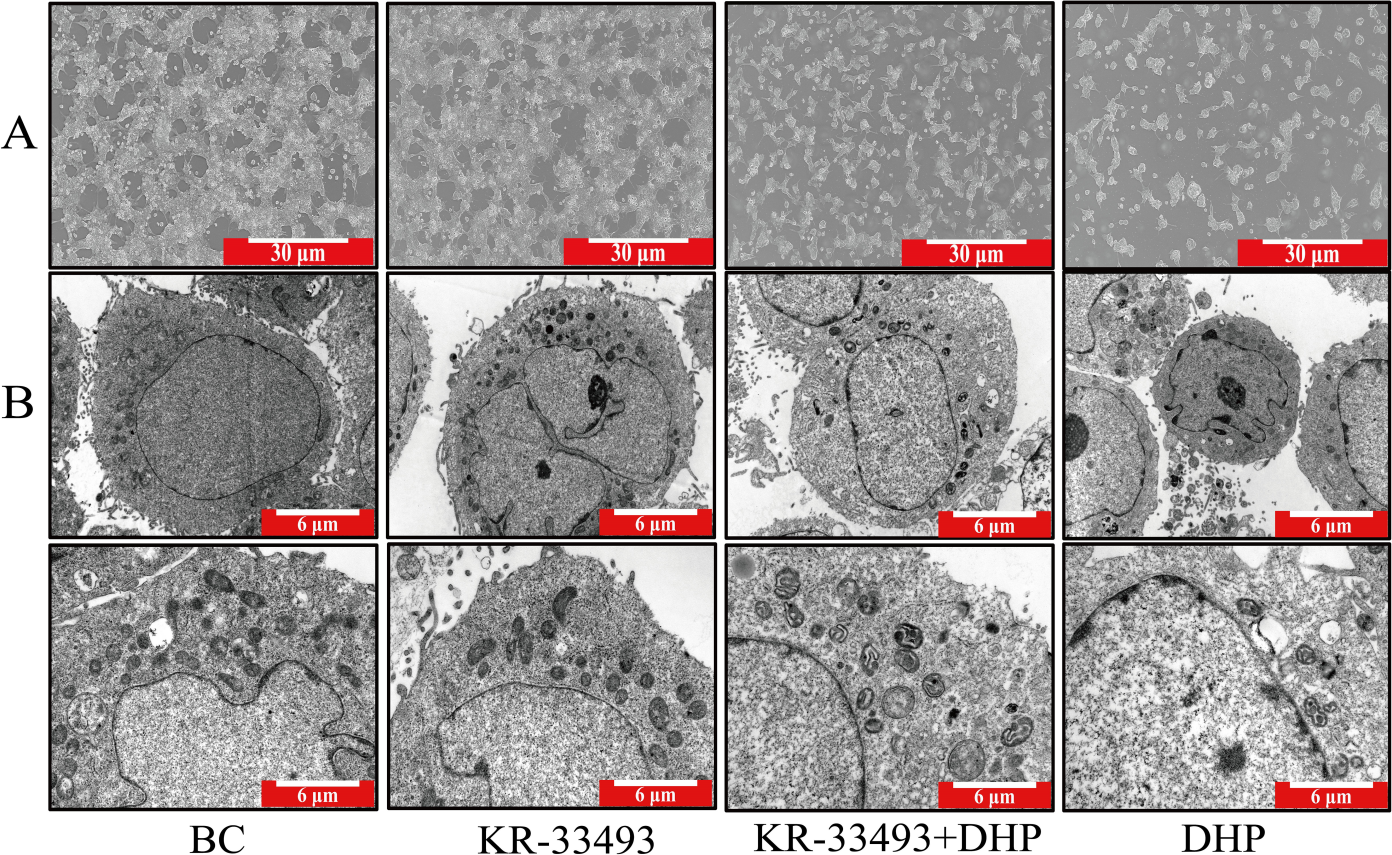
Morphology of HCT116 cells and their subcellular structures. **(A)** Light microscopy images of HCT116 cells under different treatments. The BC shows typical fusiform and clustered morphology forming cell masses. In contrast, DHP treatment (600 μg/ml for 24 h) deforms the cells disrupting the cell mass structure. The scale bar represents 30 μm. **(B)** TEM images of HCT116 cells. The BC exhibits characteristics of malignant HCT116 cells, such as irregular nuclei and deformed mitochondrial cristae indicating metabolic reprogramming to adapt to the environment. In the DHP-treated group (600 μg/ml for 24 h), cells show features of apoptosis, including chromatin condensation and reoccurrence of mitochondrial cristae, suggesting that DHP induces apoptosis. The scale bar represents 6 μm. BC, base check group; DHP, *Dendrobium huoshanense* polysaccharide.

We inspected the integral shape of HCT116 cells using an ordinary optical microscope. The results showed that under normal conditions, the shapes of individual HCT116 cells were fusiform, clumped together, and formed cell mass (**Figure 2**). However, DHP distorted this state. DHP deformed the fusiform of HCT116 cells, and the cell mass was eliminated concurrently. Moreover, we observed the subcellular structure of HCT116 cells through transmission TEM. In the normal group, HCT116 cells possessed conspicuous characteristics of pernicious tumor cells, such as erratic cell nuclei and deformation of mitochondrial crista, which prompted HCT116 cells to modify their metabolic programs to adapt to the unfavorable environment (29). However, in the DHP group, HCT116 cells emanated distinct features of apoptosis. For example, the HCT116 cells exhibited chromatin condensation and mitochondrial crista reoccurrence indicating that DHP thwarted their metabolic reprogramming and induced apoptosis.

### 3.3 DHP-induced death receptor pathway apoptosis in the HCT116 cells

**Figure 3:**
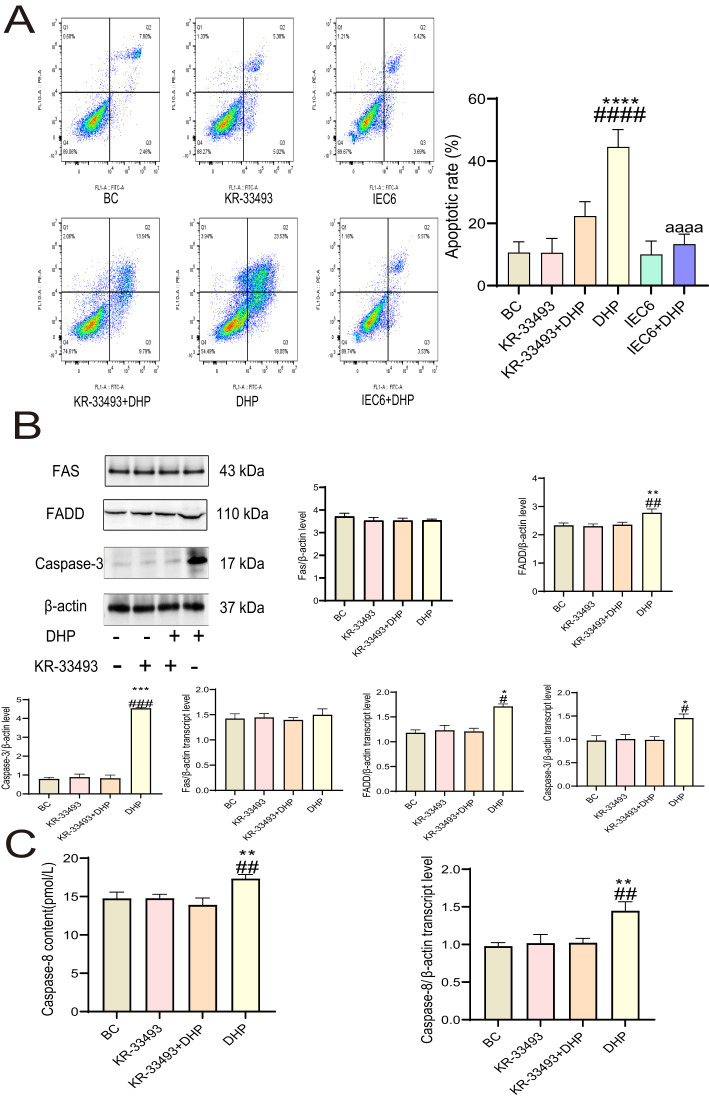
DHP induced death receptor pathway apoptosis in HCT116 cells. **(A)** The apoptotic effect of DHP on HCT116 and IEC6 cells was compared by Annexin-FITC/PI staining analysis. **(B)** The results and analysis of Western blotting and qRT-PCR of relevant proteins. **(C)** The caspase-8 content and qRT-PCR results of HCT116 cells. Data are presented as means ± SE., n = 3 (^*^p < 0.05; ^**^p < 0.01; ^***^p < 0.001; ^****^p < 0.0001 versus BC; ^#^p < 0.01; ^##^p < 0.01; ^###^p < 0.001; ^####^p < 0.001 versus KR33493 + DHP; ^aaaa^p < 0.0001 versus DHP). BC, base check group; DHP, *Dendrobium huoshanense* polysaccharide; FADD, Fas-associated death domain protein; Fas, Fas receptor; qRT-PCR, quantitative real-time polymerase chain reaction.

To further investigate the inhibitory effects of DHP on HCT116 cells, we performed Annexin-FITC/PI staining analysis. The results demonstrated that DHP caused an increased apoptosis rate of the HCT116 cells compared with that in the base check (BC) group. However, DHP did not significantly induce apoptosis in IEC6 cells compared to its effect on HCT116 cells, which further supports the selective nature of DHP’s anti-CRC activity. Notably, when HCT116 cells were pre-treated with Fas receptor inhibitor KR-33493, the DHP effects were abolished indicating that the anti-cancer mechanism of DHP may depend on Fas receptors (**Figure 3A**).

Furthermore, we aimed to demonstrate the mechanisms of how DHP activates Fas receptors; thus, we determined the content of relevant proteins at the mRNA and protein expression levels. The signal (FADD), effector (caspases-8 and -3), and ligand (FasL) proteins exhibited upregulated expression at the mRNA levels under DHP treatment implying that DHP activated the Fas–FasL signaling pathway in HCT116 cells. However, the transcript and expression levels of the Fas receptor remain constant suggesting that the activation of the Fas–FasL signaling pathway by DHP in HCT116 cells does not rely on increasing receptor content but rather on enhancing its sensitivity to ligands (**Figures 3B, C**).

### 3.4 DHP activated the mitochondrial pathway apoptosis of the HCT116 cells

**Figure 4:**
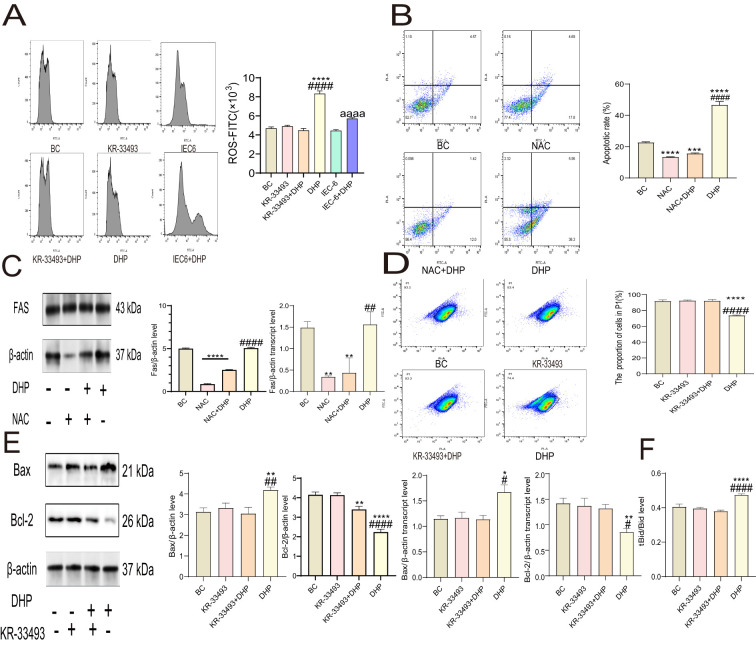
DHP activated the mitochondrial pathway apoptosis of HCT116 cells. **(A)** The DCFH-DA analysis was employed to compare the ROS-inducing effects of DHP on HCT116 and IEC6. **(B)** The Annexin-FITC/PI staining analysis. **(C)** The results and analysis of Western blotting and qRT-PCR of Fas. **(D)** The MMP analysis. **(E)** The results and analysis of Western blotting and qRT-PCR of Bax, Bcl-2. **(F)** The tBid/Bid content analysis. Data were analyzed by one-way ANOVA and expressed as mean ± SD, n = 3 (^*^p < 0.05; ^**^p < 0.01; ^***^p < 0.001; ^****^p < 0.0001 versus BC; ^#^p < 0.05; ^##^p < 0.01; ^####^p < 0.0001 versus KR-33493 + DHP in **(A)** and **(D–F)** or versus NAC + DHP in **(B)** and **(C)**; ^aaaa^p<0.0001 versus DHP). BC, base check group; DHP, *Dendrobium huoshanense* polysaccharide; Fas, Fas receptor; MMP, mitochondrial membrane potential; NAC, N-acetyl cysteine; qRT-PCR, quantitative real-time polymerase chain reaction; ROS, reactive oxygen species.

DHP is an effective drug that induces oxidative stress in CRC cells, impairs the mitochondrial structure, and triggers the mitochondrial pathway apoptosis of colon cancer cells (28, 30). Fas activation leads to the production of ROS, which in turn induces cell apoptosis; conversely, the accumulation of ROS can also trigger Fas activation (31). Herein, we concluded similarly. Our results indicate that DHP induces ROS in HCT116 cells, while the ROS increase in IEC6 cells is not as obvious compared to that in HCT116 cells (**Figure 4A**). To verify whether ROS was the cause of cell apoptosis in HCT116 cells, we added NAC to inhibit the generation of ROS. Compared with the BC group and the DHP treatment group, NAC reduced the rate of cell apoptosis in HCT116 cells (**Figure 4B**). Additionally, our qRT-PCR and Western blotting results demonstrated that NAC decreased the expression of Fas in HCT116 cells, which is consistent with previous literature reports. However, the expression of Fas in the DHP treatment group did not show a significant difference compared to that in the BC group, which aligns with the results of our previous experiments (**Figure 4C**). Moreover, the ROS-induced damage to the mitochondrial membrane structure induced by DHP impairs MMP (**Figure 4D**), downregulated the mRNA and protein expression of anti-apoptotic protein Bcl-2, and upregulated the pro-apoptotic protein Bax (**Figure 4E**). Inhibition of ROS production by NAC significantly decreased HCT116 cell apoptosis induced by DHP suggesting that increased ROS levels are indeed a critical factor for the apoptotic effects mediated by DHP. However, the nexus of the Fas–FasL signaling and mitochondrial apoptotic pathways has not been defined in the former report.

In this study, we determined the ratio of tBid to Bid, which is the signal transduction molecule between the Fas–FasL signaling and mitochondrial apoptotic pathways. The weakened activation of caspase-8 would activate the conversion of Bid to tBid and induce the mitochondrial apoptotic pathway finally (26, 27). As shown in **Figure 4F**, the results revealed that the ratio of tBid to Bid was upregulated by DHP indicating that the activation of the mitochondrial apoptotic pathway in HCT116 cells depended on the Fas–FasL signaling pathway.

### 3.5 DHP downregulated the α-2-6 sialylation modification of HCT116 cells by suppressing ST6Gal-I

ST6Gal-I is a crucial glycoside transferase that adds sialic acids through the α-2-6 linkages in the N-glycans of the membrane and secrete glycoproteins that play a protective role in different cancers (32, 33). Some research results show that high levels of ST6Gal-I can catalyze the sialylation of Fas at the terminal end. The α-2-6 sialylation of Fas inhibits the ability of Fas to induce apoptosis in colon cancer cells by blocking the binding of Fas-associated death domain proteins to the cytoplasmic tail of Fas (34).

**Figure 5:**
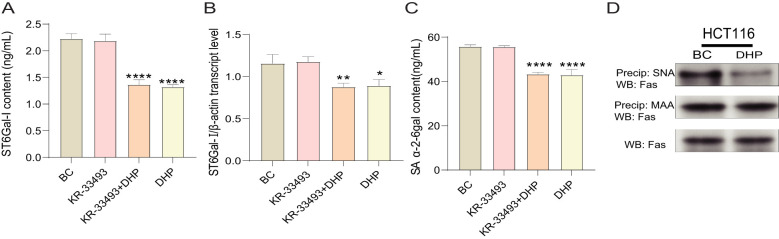
DHP downregulated the α-2-6 sialylation modification of HCT116 cells by suppressing ST6Gal-I. **(A)** The ST6Gal-I content. **(B)** The SA α-2-6gal content. **(C)** The qRT-PCR validation of ST6Gal-I. **(D)** Immunoprecipitation analysis of the effect of DHP on Fas receptor sialylation. Data were analyzed by one-way ANOVA and expressed as mean ± SD, n = 3 (^*^p < 0.05; ^**^p < 0.01; ^****^p < 0.0001 versus BC). BC, base check group; DHP, *Dendrobium huoshanense* polysaccharide; Fas, Fas receptor; MAA, *Maackia amurensis*; SA α-2-6 gal, sialic acid α-2,6-galactose; ST6Gal-I, ST6β-galactosamine alpha-2,6-sialyltransferase I; SNA, *Sambucus nigra* lectin.

Therefore, we determined the SA α-2-6 gal content and ST6Gal-I activity of HCT116 cells treated with DHP. DHP prompted a significant decrease in SA α-2-6 gal content of HCT116 cells and simultaneously inhibited ST6Gal-I activity (**Figure 5**). This indicates that the anti-cancer mechanism of DHP might involve suppressing ST6Gal-I activity and decreasing the α-2-6 sialylation modification on Fas receptors. Notably, the anti-apoptotic mechanism of α-2-6 sialylation modification on Fas receptors attenuates the sensitivity of Fas receptors to their ligands rather than downregulating their expression levels, as believed. Our previous study demonstrated that the mRNA and protein expression levels of Fas receptors in HCT116 cells treated with DHP were consistent with our initial hypothesis.

To further explore the effect of DHP on the sialylation modification of Fas, cell lysates were incubated with agarose-conjugated *Sambucus nigra* lectin (SNA), a lectin that specifically binds to α-2-6-linked sialic acids. Samples were centrifuged to selectively precipitate α-2-6-sialylated proteins, which were then immunoblotted for the Fas receptor. As illustrated in **Figure 5D**, the band representing Fas in the SNA precipitate from DHP-treated HCT116 cells appears lighter compared to that in the BC group. This suggests that, under conditions of lower ST6Gal-I expression, the α-2-6 sialylation of Fas is reduced. However, no differences were observed in the total amount of Fas protein in whole-cell lysates suggesting that altered ST6Gal-I expression affects Fas sialylation but not its overall protein expression.

Given that ST6Gal-I has two potential N-linked glycosylation sites, namely, α-2-3 and α-2-6, we next aimed to determine whether the Fas receptor is a direct target of α-2-6 sialylation. Precipitation using *Maackia amurensis* (MAA) lectin, which specifically binds to α-2-3 sialic acids, revealed that despite a decrease in α-2-6 sialylation, there was no change in α-2-3 sialylation of the Fas receptor.

### 3.6 DHP restrained the PI3K/AKT signaling pathway of the HCT116 cells and mended metabolic reprogramming

The “Warburg effect” benefits cancer cells because it offers more metabolic flux to anabolic processes such as protein glycosylation (35, 36). The classic metabolism regulation signaling pathway PI3K/AKT is significant in this phenomenon. Similarly, UDP-GlcNAc bridges cell metabolism and modification of protein glycosylation.

**Figure 6:**
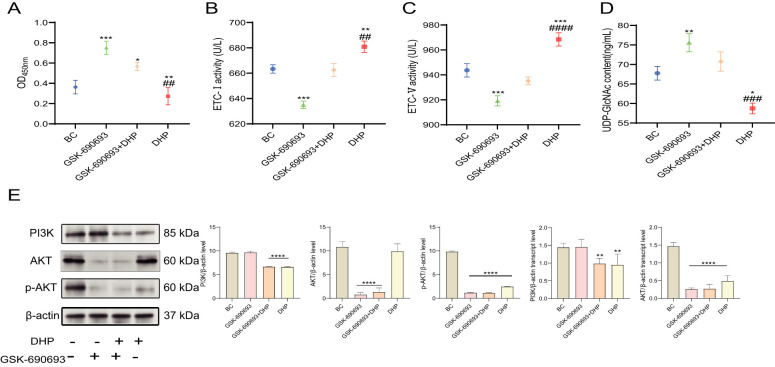
DHP restrained the PI3K/AKT signaling pathway and inhibited metabolic reprogramming. **(A)** The OD_450 nm_ of LA. **(B, C)** The ETC activity of HCT116 cells. **(D)** The UDP-GlcNAc content of HCT116 cells. **(E)** The results and analysis of Western blotting and qRT-PCR of PI3K/AKT. Data were analyzed by one-way ANOVA and expressed as mean ± SD, n = 3 (^*^p < 0.05; ^**^p < 0.01; ^***^p < 0.001; ^****^p < 0.0001 versus BC, ^##^p < 0.01; ^###^p < 0.05; ^####^p < 0.0001 versus GSK-690693 + DHP). AKT, protein kinase B; BC, base check group; DHP, *Dendrobium huoshanense* polysaccharide; ETC, electron transport chain; LA, lactic acid; OD, optical density; PI3K phosphatidylinositol 3-kinase; qRT-PCR, quantitative real-time polymerase chain reaction; UDP-GlcNAc, uridine diphosphate N-acetylglucosamine.

First, we used GSK-690693, a well-established AKT inhibitor, a well-established AKT inhibitor, at a concentration of 20 μM to investigate the effects of DHP on the PI3K/AKT pathway (37). Therefore, in this study, we investigated the LA and UDP-GlcNAc contents and ETC activity to evaluate the metabolic reprogramming of HCT116 cells and examined the PI3K, AKT, and p-AKT levels to illustrate the mechanism of DHP effect on the metabolic reprogramming of HCT116 cells. DHP initially reduced the LA and UDP-GlcNAc levels and moderately increased the ETC activity rather than rapidly inhibiting oxidative phosphorylation (OXPHOS) and mitochondrial function as anticipated. Furthermore, our observations indicate that DHP partially mitigated the effects of GSK-690693 on LA, UDP-GlcNAc levels, and ETC activity (**Figures 6A–D**). PI3K and p-AKT levels were downregulated indicating that DHP inhibits the PI3K/AKT signaling pathway in HCT116 cells. This finding further corroborates our previous experimental results (**Figure 6E**). This could be related to the ameliorating effects of DHP on metabolic reprogramming.

## 4 Discussion

The global incidence of CRC has increased significantly, and it is associated with high mortality; however, effective treatments for CRC are lacking (38, 39). Hence, developing efficacious colon cancer drugs is crucial. Polysaccharides from traditional Chinese medicines were considered a promising drug. *Dendrobium officinale*- and *Ganoderma lucidum*-derived polysaccharides reportedly possess transcendent anti-tumor properties (28, 40). Similarly, our study suggested that DHP suppressed CRC cell proliferation distinctly. However, our study implicated that the inhibitory effect of DHP on HCT116 cells is dose saturated, inconsistent with previous reports. Therefore, we concluded that the inhibitory effect of DPH on HCT116 cells is receptor mediated, as evidenced by the protective effect of KR-33493 on the HCT116 cells that resisted DHP.

The induction of apoptosis plays a crucial role in anti-tumor mechanisms. Apoptosis resistance in colon cancer cells significantly contributes to their excessive proliferation and immune escape (41, 42). In this study, we observed characteristic morphological features of apoptosis through TEM; for example, the nuclear chromatin agglutination and apoptotic body formation. Annexin-FITC/PI staining further revealed that, compared with IEC6 cells, DHP inhibited the proliferation of HCT116 cells by inducing their apoptosis program. However, the activation of the apoptotic pathway in cancer cells is regulated through various mechanisms, categorized into the intrinsic and extrinsic pathways, which comprise death receptor, mitochondrial, and endoplasmic reticulum pathways. Hence, elucidating the specific anti-tumor mechanism of DHP is crucial. In this study, we aimed to determine the anti-tumor mechanism of DHP considering the death receptor and mitochondrial pathways. In the cell viability assay, we discovered that the inhibitory effect of DHP was counteracted by Fas receptor inhibitor KR-33493. Therefore, we postulated that the anti-tumor reaction induced by DHP is Fas receptor dependent. To further verify the hypothesis, we examined the mRNA and protein expression levels of relevant proteins in the Fas–FasL signaling pathway. It is believed that the upregulation of the mRNA and protein expression levels of FADD and caspase-8 indicates the activation of the Fas–FasL signaling pathway and that the effect can be reversed by KR-33493. However, the mRNA and protein expression levels of Fas receptors were constant implying that DHP activates the Fas–FasL signaling pathway by strengthening the sensitivity of its receptors rather than upregulating their levels.

In addition, according to previous reports, DHP induces the mitochondrial apoptotic pathway of CRC cells by upregulating the ROS content and impairing mitochondrial function, activating the mitochondrial apoptotic pathway of colon cancer cells (28, 43). Among the regulatory mechanisms of apoptosis, DHP has demonstrated significant effects by modulating ROS levels and interacting with the PI3K/AKT signaling pathway. Research indicates that DHP can enhance ROS production, thereby activating downstream signaling cascades. The elevated intracellular ROS concentration triggers an oxidative stress response, which in turn influences cell survival. Additionally, DHP inhibits the activation of the PI3K/AKT signaling pathway, thereby limiting the transmission of pro-survival signals and promoting apoptosis (44). The inhibition of AKT results in increased ROS production, which subsequently impairs the MMP and activates the mitochondrial apoptosis pathway (45). Similarly, in this study, we found that DHP exerted similar effects on HCT116 cells. The elevated ROS content and declined MMP induced by DHP triggered the mitochondrial apoptotic pathway of HCT116 cells. However, in this study, the levels of the tBid/Bid ratio of HCT116 cells subjected to DHP were slightly increased indicating the deviation from the death receptor to the mitochondrial apoptotic pathway. We concluded that DHP is a potent Fas–FasL signaling pathway agonist and activates the mitochondrial apoptotic pathway by suppressing the activation of caspase-8 and triggering the Bid and tBid conversion.

Studies have demonstrated that polysaccharides are potent Fas–FasL signaling pathway agonists, which exert transcendent anti-tumor effects (46, 47). Therefore, investigating how polysaccharides activate the Fas–FasL signaling pathway is essential. Considering that Fas receptors are highly glycosylated and that the glycosylated modification of Fas receptors is significant in their activation and inhibition (23, 48), the mechanism by which DHP activates the Fas–FasL signaling pathway might involve modifying the glycosylation pattern of Fas receptors. It has been reported that the excessive α-2-6 sialylation on Fas receptors in colon cancer, catalyzed by ST6Gal-I, thwarts the receptor oligomerization and signaling induced by FasL and anti-CD95 antibody. Therefore, we examined the SA α-2-6 gal content and ST6Gal-I activity of HCT116 cells treated with DHP. The results demonstrated that DHP induced significant decreases in SA α-2-6 gal content and ST6Gal-I activity. Immunoprecipitation analysis confirmed that high expression of ST6Gal-I led to increased α-2-6 sialylation of Fas suggesting that DHP reverses the abnormal α-2-6 sialylation on Fas receptors, thereby modulating their sensitivity to specific ligands. This finding explains why the levels of Fas receptors remained unchanged before and after DHP treatment. Additionally, in this study, we found that DHP induced a significant increase in ROS levels in HCT116 cells, and reducing ROS levels could markedly decrease Fas expression, which is consistent with previous reports in the literature (49, 50). Concurrently, although DHP activated the Fas–FasL signaling pathway in HCT116 cells, the transcription and expression levels of the Fas receptor did not change. This suggests that DHP regulates Fas by enhancing its sensitivity to FasL and promoting Fas sialylation.

By combining this finding with the metabolic regulatory activity of polysaccharides in certain metabolic diseases, such as diabetes (51–53), we extrapolated that the essential anti-cancer mechanism of DHP involves restoring the disturbance of cancer cell metabolism. The “Warburg effect” is an extensively studied cellular metabolic reprogramming pattern. This phenomenon enables cancer cells to exploit increased anabolic flux, thereby facilitating aberrant glycosylation modifications in specific cancer cells, such as those found in colon cancer (54). The molecular mechanisms of “Warburg effect” are complex; however, the PI3K/AKT signaling pathway was identified as a crucial factor in the development of “Warburg effect,” as its abnormal activation induced the metabolic switch between OXPHOS and glycolysis (55). UDP-GlcNAc is a pivotal glycosyl donor amid the protein glycosylation. The generation of ROS appears to be a critical link between metabolic reprogramming and mitochondrial apoptotic pathways. Elevated ROS levels can promote mitochondrial dysfunction and apoptosis (56), while also influencing metabolic pathways by modulating the activities of key enzymes involved in energy metabolism. Specifically, increased ROS not only triggers apoptosis via the mitochondrial pathway but also signifies a shift in cellular metabolism. In conclusion, our study provides compelling evidence that DHP induces apoptosis through the mitochondrial pathway while concurrently modulating associated metabolic processes.

In this study, we investigated the LA content and ETC activity of HCT116 cells in the early stage of treatment with DHP. The data implied that within 12 h after DHP administration, the polysaccharide induced a decreased LA content and a slight upregulation of ETC activity. The cell viability assay showed that the anti-proliferative effects of DHP were negligible at 12 h. In addition, we observed that DHP caused an increased synthesis of UDP-GlcNAc, a vital glycosyl donor in the process of protein glycosylation. When the genes of the PI3K/AKT pathway and their phosphorylated proteins undergo changes, it indicates that the pathway has been affected (57, 58). DHP inhibited the PI3K/AKT signaling pathway of HCT116 cells and altered its development of metabolic reprogramming causing a decline in the anabolic metabolic flux. Our findings suggest that the mitochondrial activation of apoptosis induced by DHP is closely linked to the metabolic reprogramming observed in HCT116 cells. Specifically, the elevation of ROS not only triggers apoptosis via the mitochondrial pathway but also reflects a shift in cellular metabolism. This indicates that metabolic reprogramming and apoptosis are intricately interconnected processes. Subsequently, the deficient UDP-GlcNAc and suppressed ST6Gal-I mitigated the aberrant glycosylation pattern of HCT116 cells causing decreased α-2-6 sialylation and restored function of the Fas receptors inducing the apoptosis of HCT116 cells.

**Figure 7:**
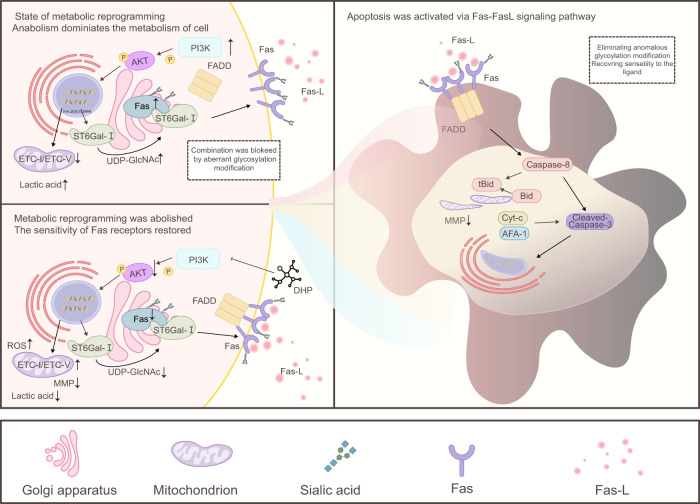
Mechanism of DHP-mediated apoptosis in HCT116 cells. In the state of metabolic reprogramming, cell metabolism shifts primarily toward anabolism, and DHP promotes this process by inhibiting the PI3K/AKT signaling pathway. Downstream of AKT, it regulates ST6Gal-I in the cytoplasm and ROS in the mitochondria. Specifically, AKT inhibits ST6Gal-I by downregulating the sialic acid modification of Fas, thereby enhancing Fas ligand sensitivity and activating downstream caspase-8. This leads to the cleavage of Bid into tBid, which translocates to the mitochondrial membrane resulting in a decrease in MMP. Simultaneously, AKT promotes ROS production, and high levels of mitochondrial ROS further damage MMP. Together with the aforementioned Fas receptor pathway, this results in a significant reduction in MMP promoting the leakage of proteins such as cytochrome c (Cyt-c) and apoptotic protease-activating factor-1 (APAF-1), activating caspase-3, initiating the caspase cascade, cleaving nuclear DNA, and ultimately inducing apoptosis. AKT, protein kinase B; DHP, *Dendrobium huoshanense* polysaccharide; ETC, electron transport chain; FADD, Fas-associated death domain protein; Fas–FasL, Fas receptor–Fas ligand; ROS, reactive oxygen species; SA α-2-6 gal, sialic acid α-2,6-galactose; UDP-GlcNAc, uridine diphosphate N-acetylglucosamine.

We demonstrated that DHP induced the death receptor and mitochondrial apoptotic pathways of HCT116 cells potently (**Figure 7**). Despite being a large water-soluble polysaccharide, DHP significantly inhibits the PI3K/AKT signaling pathway in HCT116 cells consistent with the hypothesis that DHP may block AKT phosphorylation, thereby reducing downstream AKT activation. This inhibition is supported by the observed decrease in key metabolic intermediates, such as UDP-GlcNAc, which contributes to metabolic reprogramming. The inhibitory effect of DHP on HCT116 cells is associated with the inhibition of Fas receptor α-2-6 sialylation, restoration of its function, and enhanced sensitivity of the Fas receptor to its ligand FasL. This allows lower concentrations of DHP to effectively induce apoptosis. Despite these limitations, this study has several strengths. First, we used a novel approach to investigate the effects of DHP on colorectal cancer cells, which has not been previously reported. Second, we identified a novel molecular mechanism by which DHP exerts its anti-tumor effects, which may provide new insights into the development of novel therapeutic strategies for colorectal cancer. Third, our study provides evidence that DHP can be used as a potential therapeutic agent for colorectal cancer, which warrants further investigation in clinical trials.

## Data Availability

The raw data supporting the conclusions of this article will be made available by the authors, without undue reservation.
